# Religious adaptation of a parenting programme: process evaluation of the Family Links Islamic Values course for Muslim fathers

**DOI:** 10.1111/cch.12228

**Published:** 2015-02-04

**Authors:** J. Scourfield, Q. Nasiruddin

**Affiliations:** ^1^ Cardiff School of Social SciencesCardiff UniversityCardiffUK; ^2^ Aga Khan UniversityKarachiPakistan

**Keywords:** cultural adaptation, fathers, intervention, Islam, parenting

## Abstract

**Background:**

Amid concern about the reach and inclusivity of parenting interventions, attempts have been made to culturally adapt programmes for specific ethnic or linguistic groups. This paper describes a novel approach of the religious adaptation of a parenting programme, namely the Family Links Islamic Values course.

**Methods:**

A small‐scale qualitative process evaluation was conducted on one Family Links Islamic Values course for Muslim fathers in the South of England in order to describe the intervention as implemented and its theory of change, as well as the acceptability of the programme to the participants. The data consisted of 13 semi‐structured interviews (10 with parents and three with staff), 25 h of observation and reading of programme manuals.

**Results:**

A logic model is presented to describe the theoretical basis of the intervention. The programme was highly acceptable to fathers who valued the integration of religious teachings and were generally very positive about their experience of attending the course. Post‐course interviews with both fathers and mothers mentioned some positive changes in fathers as a result of their attendance.

**Conclusions:**

It is important to be responsive to the needs of some British Muslims for religiously credible interventions. This small‐scale process evaluation needs to be followed by a robust evaluation of programme outcomes for parents and children.

## Introduction

### Cultural adaptation of parenting programmes

There is considerable interest among policy makers and practitioners in preventative ‘early intervention’ programmes to improve children's well‐being. These programmes are usually focused on changes to parenting style, and some approaches are quite strongly evidence based (Barlow *et al*. 2011; Furlong *et al*. 2013). Despite a generally optimistic climate surrounding early intervention (some prefer ‘early help’), there has been some concern expressed about the extent to which parenting programmes are inclusive (Davis *et al*. 2012). One aspect of this lack of inclusiveness relates to minority ethnic populations, with concern that they have not always been included in the development of evidence‐based interventions and calls for those interventions with a good evidence base for majority populations to be culturally adapted for specific cultural groups (Castro *et al*. 2010).

This is a complex terrain with many pitfalls such as assuming ethnic homogeneity, and there is an ongoing dilemma about how to balance cultural relevance with fidelity to the intervention's core principles (Castro *et al*. 2010). The assumption of cultural adaptation is that programmes will be more effective if culturally relevant to participants. Castro and colleagues (2010) summarize the evidence on the effectiveness of cultural adaptation of psychological interventions, noting that there is no clear consensus, with more and less optimistic results found in the reviews by Griner and Smith (2006) and Huey and Polo (2008), respectively. Griner and Smith's review was wider in scope and more inclusive of a range of interventions. They found stronger effects for intervention participants who were less accultured to their majority context, e.g. not fluent in the dominant language.

There are many different levels at which programmes can be adapted. Cultural adaptation can be surface – i.e. addressing aspects of culture such as language, food and dress – or deep – i.e. serious attention is paid to ‘cultural, social, historical, environmental, and psychological factors that influence the health behaviors of members of a targeted population’ (Castro *et al*. 2010, p. 216, with reference to Resnicow *et al*. 2000). Falicov (2009) has identified three levels of depth in the adaptation of programmes. The strongest incorporation of a distinct cultural context is what Falicov terms ‘culturally informed’ interventions. These put the cultural context to the fore, possibly prioritizing it over fidelity to the original intervention. A slight weaker modification is ‘culturally adapted’ interventions where much of the original intervention is maintained. Weaker still is where interventions are ‘culturally attuned’, i.e. some minor changes are made to remove any obviously off‐putting elements of the original intervention and improve accessibility.

Although a religious world view may form part of the cultural context that programme adaptations try to allow for, we are not aware of examples of research on the deliberate religious adaptation of a parenting programme. Previous adaptations have focused on ethnic and linguistic communities rather than religious ones. For a parenting programme based on psychological theory to be adapted for a specific religious group is therefore a novel approach. The intervention that is the focus of this study was adapted for Muslims of diverse ethnic background rather than for a specific ethnic–linguistic group. This reflects how many Muslims see themselves, prioritizing religious identity over ethnic or national identity (Roy 2004).

### Islamic social welfare

Islam is a growing religion worldwide (Sutton & Vertigans 2005) and there are substantial Muslim minorities in many European countries. For example, in England and Wales, the Muslim population was 2.7 million in 2011, having grown between the censuses of 2001 and 2011 from 3% of the population to 5% [Office for National Statistics (ONS 2013)]. There have been some attempts to provide Islamically sensitive social work services developed from a faith‐based position. The existing literature tends to describe an Islamic approach to services in theory, but there is lack of evidence about how this plays out in practice (one exception is Warden 2013).

Scourfield and colleagues (2013) have recently argued, with reference to research with British Muslim families, that although there is course diversity in the Muslim population, there is also a broad agreement about some aspects of child‐rearing that have implications for family welfare services. These aspects include a strongly monotheistic world view and traditions of formal religious education. One of their conclusions is that parenting programmes may need to be offered from a faith perspective if Muslim parents are to be successfully engaged.

Adaptation of parenting interventions for Muslim families is beginning to happen. Approachable Parenting (http://www.approachableparenting.com) is a parenting programme that uses mainstream psychological principles but also a faith perspective, and is specifically designed for Muslim parents rather than being an adaptation of a pre‐existing secular programme. In Falicov's (2009) terms this is probably a ‘culturally informed’ approach. The Strengthening Families Programme (http://www.mystrongfamily.co.uk) has an adapted version for Muslim families, which initial conversations with practitioners suggest is being fairly minimally revised for Muslim families in the United Kingdom, with an emphasis on fidelity to the original programme. It would therefore probably fit with what Falicov (2009) terms a ‘culturally attuned’ approach.

### The Family Links Nurturing Programme

The Family Links Nurturing Programme is a British adaptation of a programme originally devised by Steven Bavolek in the United States. It can in theory be used as a universal programme, although in practice it is used with families in need, at least in deprived areas. The theory of change framework (Colebrook Centre and Family Links, 2014a) states that the main focus is on ameliorating parents' emotional and cognitive problems, which facilitates the increased use of positive parenting skills, resulting in children being calmer, happier and more co‐operative. The intervention is based on psycho‐educational and cognitive–behavioural approaches to learning. The UK programme is based on four main constructs: empathy, positive discipline, age‐appropriate expectations and self‐awareness, with various exercises based on these. There is a stronger emphasis than in some other programmes on the well‐being of the parent as opposed to a focus solely on interactions with children. A course runs for 10, 2‐h sessions that are all group based and interactive. There is use of role play and trying out strategies at home in‐between sessions.

This Family Links Islamic Values course follows this same model, but with accompanying religious texts to support the core programme messages as will be explained in the Findings section. It is available for all parents, but single‐sex groups are offered as this is in keeping with Islamic traditions. Only the fathers' group was studied in this small‐scale study, which formed part of a programme of research on social interventions for fathers.

## Research methods

The study was a process evaluation designed to explore intervention acceptability to both fathers and their wives, to describe the intervention as implemented, including the practical working out of the religious adaptation, and to describe its theory of change. This was a small‐scale qualitative study. Data collection consisted of studying the programme manuals, about 25 h of participant observation of one course in the South of England, focusing on intervention delivery and participants' interaction before and after group meetings, and 13 semi‐structured interviews. Interview participants consisted of five fathers who had completed the course we observed (seven had started, with two dropouts), five women who were the wives of the men interviewed and had themselves attended the Family Links programme in a mothers' group, and two staff members, one of whom was a senior staff member with Family Links and the other of whom (interviewed twice) was heavily involved in adapting the programme for Muslim parents (‘Khadija’). Interview participants are identified in the paper either by job role or with pseudonyms.

All observation and client interviews were conducted by Qurratulain Nasiruddin through the medium of Urdu. Field notes were written in English. Interviews conducted in Urdu were translated into English by the researcher. The only aspect of intervention content that could not be observed was the session on sexuality, which was a men‐only session, so the researcher and female facilitator left the room. The three staff interviews were conducted in English by Jonathan Scourfield. Of the five fathers interviewed, four had Pakistani ethnic origin and one Indian. One father was referred as a child protection case; two had been identified as in need but not meeting the threshold for services; a further two were self‐referred and their wives had already completed the programme. Family size ranged from two to six children. Men were unemployed or self‐employed in retail or taxi driving. Two could speak English and three could not, but all five understood Urdu and Punjabi well. One of the facilitators was not fluent in Urdu, so there was primarily as support for Khadija.

Data were thematically analysed using what Coffey and Atkinson (1996) term a ‘code‐and‐retrieve’ approach. Data were initially sorted (using Microsoft Word) under thematic headings reflecting the purpose of the process evaluation, and then a commentary was developed for each theme in order to present an overview of responses.

## Findings

### Developing and delivering the Islamic Values course

The Family Links Islamic Values course was developed by Khadija, a family support worker. Khadija was very enthusiastic about the Family Links Nurturing Programme after being trained to facilitate it but was concerned about lack of take‐up from Muslim parents.

It was really difficult to engage Muslim parents in parenting programmes because they wouldn't get engaged, and they would say ‘this is a kind of a programme which is just for British way of living, it is nothing to do with our belief system’. (Interview with Khadija)

Khadija herself saw the Family Links Nurturing Programme as completely compatible with Islam. A religion–culture distinction is commonly made by Muslims in the West (Roy 2004; Bolognani & Mellor 2012), and it was felt that Muslims often struggle with this distinction in relation to parenting. Khadija's view was that even some aspects of the programme that are not usually associated with Islam, because of the cultures of Muslim countries rather than the teachings of religious texts, are in fact fully compatible with Islam. The examples she gave in this regard were openness about sex, children's rights and women's rights (see Giladi, 2014 on the former). Discussion of sex was described as a challenge because Muslim parents often think it is shameful, but as not in fact un‐Islamic if there are some limits, such as that discussion of body parts should take place in single‐sex groups. The whole course is in fact run via single‐sex parenting classes as this was thought to be more acceptable to the target population. The fathers' group is facilitated by Khadija herself and a male Islamic counsellor because of a dearth of trained men. Khadija told us that this was not ideal but it was a necessary compromise to make the group viable. When sex is discussed in a session, Khadija absents herself some of the time, returning for the discussion of keeping children safe from sexual harm.

The process of adaptation was that Khadija herself identified texts from *Qur'an* and *Hadith* (sayings of the Prophet Muhammad) that gave an Islamic justification for the messages of each programme session; this approach was piloted with a group of Muslim mothers who were studying the *Qur'an*; the content was then checked with Muslim scholars, a local *Sharia* council and the Muslim College of London. This process took a year. Before publishing the Islamic Values booklet for parents, which contained these religious texts in English translation, the scholar Tariq Ramadan was approached to write the foreword for the booklet. The programme is now being rolled out through training of women volunteers recruited via the UK Islamic Mission. These are lay people rather than professionals. Programmes have tended to run through the medium of a community language (e.g. Urdu or Punjabi). So although the programme is designed to be suitable for a multi‐ethnic Muslim audience, in practice the groups tend to be ethnically specific. In the Family Links staff member's view, it tends to be ‘less integrated’ parents who attend because this group would be less likely to attend a non‐Islamic programme. Care was taken to develop a programme suitable for more or less all schools of thought within Islam. As Khadija put it:

There are 72 sects in Islam and I want to make sure that everything that we are discussing and talking is acceptable for everybody. (Interview with Khadija)

**Figure 1:**
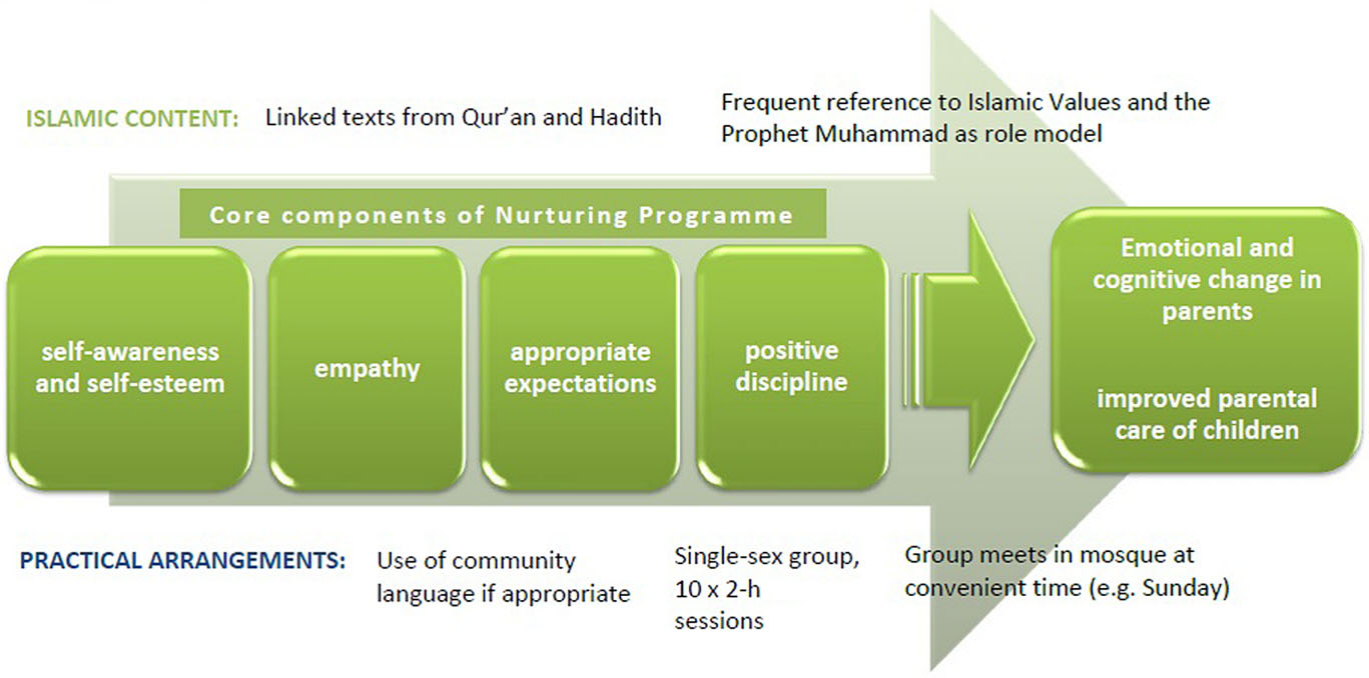
Logic model diagram for the Family Links Islamic Values course.

The adapted programme does not involve any elements being removed from the Nurturing Programme. The intention is to preserve all the core elements of the original (secular) programme, but to package them for a Muslim audience with Islamic teachings that are compatible. Logic models already exist for the Nurturing Parenting family of programmes (Bavolek n.d.) and the Family Links Nurturing Programme (Colebrook Centre and Family Links, 2014b). Figure 1 shows a visual representation of the main elements of a logic model for the Islamic Values course, with religious teachings bolstering the core themes of the original programme and practical arrangements, such as timing, location and language, increasing the programme's accessibility.

The delivery stuck quite closely to the manual, with all main programme elements present as well as the religious framing. Inevitably, less time was spent on activities specified in the manual as would be the case for the regular Family Links programme as the group sessions take up the same amount of time as the regular programme, but some of this time is needed for religious framing. Time constraints did not allow for all the materials in the Islamic values booklet to be used, just as not every detail in the Family Links manual was used. So, for example, the detail of the time out strategy was skated over and session 4 had relatively little Islamic content, with religious texts read out for one of the session's themes only. Despite the constraints of time, there was little evidence of dilution of the core programme elements, even with the course lasting only 9 weeks instead of 10 because of the availability of some of the fathers.

As noted earlier, the course introduces perspectives and strategies in relation to parenting not because they are Islamic as such but because they are core to the Family Links Nurturing Programme. However, participants in the Islamic Values course are given examples from the life of the Prophet Muhammad to demonstrate that the proposed strategies do not contradict Islamic principles. Examples were that the importance of parents modelling behaviour for children was supported by the story of the Prophet's grand children who demonstrated the correct way of ablution rather criticizing a man who was not performing it correctly. The theme of punishment and rewards was illustrated by the example of Muhammad forgiving others and showing generosity. Equally, his approach of consulting family members and neighbours rather than imposing his decision was used to underline the importance of involving family members in decisions.

Although the programme was developed for Muslim parents of any ethnic background, the particular course we observed was attended by four Pakistanis and one Indian father. The shared ethnic background of most men meant that there was also discussion of Pakistani culture in comparison with the United Kingdom. The programme was conducted in the Urdu language. This suited the fathers well, but the male co‐facilitator struggled with this a little, understanding Urdu but not speaking it well.

### Acceptability of the programme

The fathers were very positive about the programme in general, making some very supportive comments about what can be gained from attendance:

The purpose of the programme is so good that nothing else could make it better. It's perfect. (Mr Khan)

This is a very good programme if you see it is very helpful for the development of a person as a human being not as a parent. This programme makes you a good human being. (Mr Hussain)

This programme is very helpful. I recommend this programme to most of the family members. I am not only talking about Asian people. Everybody can take it. It's a very good course. Especially since the government is giving funds and it's free of cost as well. (Mr Malik)

The fathers were very positive about the facilitators, and Khadija in particular, acknowledging that she took the lead in the running of the programme. None of them seemed to see it as problematic to have a female facilitator. Specific aspects of the course that fathers spoke positively about were the timing of the meetings on a Sunday, meaning it could fit around work patterns; the interactive nature of the sessions, making them ‘fun’; and the fact that the material gets people thinking, rather than being about ‘giving information’. There were also positive comments about the relationships between the fathers built up in the group.

The fathers valued the Islamic content. Mr Khan noted that it is important to follow the ‘laws and regulations of England’ but it is also important that ‘our values and principles in families should be in accordance to Islam’. The programme's approach of emphasizing the compatibility of the parenting advice with Islamic teachings was appreciated as expressed in the following interview excerpt.

If you are talking to Muslims, it is very very good. We Muslims always refer to Qur'an, Hadiths and its interpretations like Sahih Bukhari (…) They provided teachings from Qur'an and Hadiths on feelings like how do you feel? what to do when you feel upset? It is very good actually. I would say that you could add more but not remove. (Mr Malik)

Like Mr Malik, Mr Hussain also thought that the Islamic material ‘should be increased (…) it's better to learn more about Islamic content’.

A couple of the fathers also mentioned the appreciation of ethnic culture, as well as faith. Mr Malik said ‘Khadija has an Asian background so she will understand our culture and she can give us better examples’. Mr Khan spoke about not knowing previously how to express some ideas that were discussed on the course and learning new language even if the concepts were not novel to him:

Since we are Pakistani, we do not have much knowledge and we lack exact terms to communicate our thoughts and ideas. For example, I used to do ‘time out’ but I never used this term as I didn't know. Thus, social services thought that I should join this programme to learn about parenting. When I started the programme, I realised that I have been doing some of things but I also learnt additional things such as empathy and child psychology. I used to do before as well but I never used to say. Hopefully, I am now in a position to explain everyone. (Mr Khan)

The fathers were asked if they had ideas for improving the programme and relatively few were expressed, which in large part reflected the extent to which they were positive about their experience (Mr Siddiqui said ‘I will just say that everything is perfect’).

As noted earlier, two fathers wanted more Islamic content. One father commented on the need for all English used in the group to be translated into Urdu. Two fathers had ideas about diversifying the style and medium of learning. Mr Ibrahim recommended using video clips or ‘some visual thing to see’ and a ‘mini test for fun’ to aid memory retention. Mr Malik suggested ‘more interaction’ and ‘team‐building exercises’. He mentioned that fun exercises were used as ice breakers but that the use of these had declined. In observing the group we noticed that men rather came alive when such practical exercises were used. Mr Ibrahim also thought a larger group size would ‘make it more interacting and more fun’.

All interviewees were asked about changes achieved as a result of the fathers' group. The women interviewed spoke in mostly optimistic terms of changes in the fathers' attitude and behaviour towards themselves and the children, e.g. less anger being expressed and more empathy. Some had seen positive changes in their children following the parents' attendance at the course. There were also some limitations in the change achieved, however. Mrs Hussain said her husband had yet to control his anger or learn to trust her.

As would be expected, since the programme addressed parenting, the fathers spoke mainly about their behaviour in relation to their children, when asked about the impact of attending the programme. Each of the five fathers highlighted a different issue that had been the main point of learning for him. This list included learning ‘self‐power to handle family matters’ and implementing behaviour management strategies, co‐operation between husband and wife about getting children ready for school, leaving the room when angry, controlling emotions, displaying empathy and connection to children and spending more time and sharing more concerns with wives.

Despite these encouraging testimonies, there were also some examples of limited insight. Khadija noted that the fathers tend to start off by expecting the rest of the family to change and it takes time to ‘convince them it's you, you who have to change’. One example of this that we observed was that one participant had introduced a kindness chart, as recommended in the programme, but expected his wife and children to complete the chart rather than taking part himself. Also, one of the fathers explained in his post‐programme interview that his wife was the sole problem in terms of parenting.

## Discussion and conclusion

Muslim fathers could be seen as doubly alienated from conventional parenting interventions, insofar as programmes may be seen as unsuitable both because they are secular and because they are dominated by mothers. Any approach that successfully engages them is therefore to be welcomed and more work is needed to assess its effectiveness. The Family Links Islamic Values programme appears to be highly acceptable to Muslim fathers and their wives who also attended a parallel programme. There are, however, limitations to what conclusions can be drawn from a small‐scale study of only one group, with data only from programme staff and parents. It can be expected that programme staff will be positive about their work and this in itself is not evidence for the usefulness of the programme. Although observation adds depth to a qualitative study, it may be that the researcher was more identified with the programme as a result of her attendance, leading to socially desirable positive viewpoints being expressed in the interviews. Also, the views of the men's wives may well have been shaped more by their own experience of attending the programme than by any change in their husbands.

It may be that a religiously adapted programme is especially effective for parents such as the fathers attending the course we observed because they are relatively unaccultured to the UK context, with some not speaking English (Griner & Smith 2006). There is no suggestion that the programme is only suited to high‐risk families as the course we observed was attended by parents across the spectrum of need, including those who self‐referred. In terms of Falicov's (2009) three levels of cultural adaptation, the Family Links Islamic Values programme would seem to fit in the middle level, that of a ‘culturally adapted’ programme, as the core elements of the original secular programme are maintained but framed with compatible Islamic teachings. A challenge for the programme is fitting in enough of the material from the original secular programme while also keeping space in the sessions for the religious content.

The Family Links Islamic Values course is an example of the adaptation of a social intervention for a religious group rather than an ethnic group. Research is now needed into the effectiveness of such an approach. It would be important in the future to study the outcomes of programme attendance in terms of parent and child well‐being. This would ideally involve more objective measures than parents' self‐report as well as a robust sample size. If possible, it would be very useful to compare Muslim parents attending a non‐adapted secular parenting course with parents attending a course that includes Islamic teachings, as well having as a control group that receives only routine services.

## Key messages

- Parenting interventions need to be accessible to diverse populations
- Muslim fathers can be doubly alienated from family welfare services, if these services are secular and dominated by mothers
- Unlike most examples of cultural adaptation, the Family Links Islamic Values course has been adapted for a (multi‐ethnic) religious group, rather than a specific ethnic or linguistic group
- The adaptation involves keeping the original core programme intact but overlaying it with religious teachings that are relevant and compatible
- A small‐scale study of the programme for fathers found it to be highly acceptable to both fathers and mothers

## Funding

The research was funded by the Economic and Social Research Council as part of a fellowship for Jonathan Scourfield (RES 070‐27‐0040). Some additional research expenses were provided by Family Links.

## Conflict of interest

As noted above, some additional research expenses were provided by Family Links.
